# Selective Vein Graft Cold Cardioplegia and Warm Reperfusion to Enhance Early Recovery in Patients with Left Ventricle Depression Undergoing Coronary Artery Surgery

**DOI:** 10.3390/jcdd12060222

**Published:** 2025-06-12

**Authors:** Pasquale Totaro, Martina Musto, Eduardo Tulumello, Antonella Degani, Vincenzo Argano, Stefano Pelenghi

**Affiliations:** 1 Cardiac Surgery, IRCCS Foundation Hospital San Matteo, 27100 Pavia, Italy; martina.musto01@universitadipavia.it (M.M.); a.degani@smatteo.pv.it (A.D.); s.pelenghi@smatteo.pv.it (S.P.); 2 Cardiac Surgery, University Hospital P Giaccone, 90127 Palermo, Italy; e.tulumello@unipalermo.it (E.T.); vargano@hotmail.it (V.A.)

**Keywords:** myocardial ischemia, coronary artery, bypass aorto-coronary, cardioplegia, myocardial protection

## Abstract

**Background:** Antegrade root cardioplegia remains the most popular strategy for myocardial protection during coronary artery bypass graft (CABG) performed with cardiopulmonary bypass (CPB) and aortic cross clamp. In patients with depressed left ventricular function, however, especially if associated with severe multiple coronary stenosis, increased pharmacological and/or mechanical support in the early post-CPB period is often required to support left ventricular recovery. In this study, we analyzed the results of a myocardial protection strategy that includes selective infusion of cardioplegia through each venous graft followed by warm reperfusion distal to each coronary anastomosis until complete removal of the aortic clamp (total antegrade cardioplegia infusion and warm reperfusion = TAWR) to improve early postoperative recovery in patients with depressed left ventricular function undergoing multi-vessel CABG. **Methods:** Out of 97 patients undergoing CABG using the TAWR strategy for myocardial protection, 32 patients presented with depressed left ventricle function (EF < 40%) and multi-vessel coronary diseases requiring ≥2 vein grafts and were enrolled as Group A. Combined primary outcomes and postoperative early and late left ventricle recovery (including spontaneous rhythm recovery, inotropic support and postoperative troponin release) were analyzed and compared with those of 32 matched patients operated on using standard antegrade root cardioplegia and limited warm reperfusion through LIMA graft (SAWR) enrolled as Group B. **Results:** Two patient died in hospital (in-hospital mortality 3.1%) with no statistical differences between the two groups. In Group A 27 patients (90%) had spontaneous recovery of idiopathic rhythm compared to 17 (53%) in group B (*p* = 0.001). Early inotropic support was required in nine patients (28%) of group A and seventeen patients (53%) of group B (*p* = 0.041). Furthermore, in eight patients (25%) of group A and seventeen (53%) of group B (*p* = 0.039) inotropic support was continued for >48 h. **Conclusions**: The TAWR strategy seems to significantly improve early postoperative cardiac recovery in patients with left ventricle depression undergoing multi-vessel CABG, when compared with SAWR strategy and could therefore be considered the strategy of choice in this subset of patients.

## 1. Introduction

Although the introduction of percutaneous angioplasty with stent deployment [1] has represented a milestone in modern treatment of ischemic heart disease, coronary artery bypass grafting (CABG) still remains the option of choice in selected cases due to both anatomical and prognostic reasons [2,3,4]. When indicated, CABG is still mainly performed using cardiopulmonary bypass pump (CPB) [5,6] and myocardial protection during aortic cross-clamping (and myocardial ischemia) is surely the key issue to achieving a satisfactory postoperative outcome, especially in patients with preoperative depressed left ventricle function. To reduce myocardial oxygen demands, achieving protective diastolic arrest represents the most frequently used myocardial protection strategy combined with the use of cardiopulmonary bypass. Since 1955 when cardioplegia was introduced [7], several advances have been achieved regarding both the solution of cardioplegia itself and the infusion methods, and several studies have been focused on the comparison of such different strategies in order to optimize perioperative myocardial protection during cardiac surgery procedures [8,9,10,11,12,13,14,15,16,17]. In CABG procedures one of the most frequent protocols of myocardial protection includes cardiopulmonary bypass, aortic cross clamp and cardioplegic-induced diastolic arrest by mean of antegrade infusion (via aortic root) of a loading dose of either crystalloid or blood cardioplegia, followed by a dose of systematic maintenance dose every 15–30 min until aortic cross-clamp removal. Proximal saphenous vein graft anastomoses are completed both during the aortic cross-clamp period or, following cross clamp removal, during a second period of partial aortic cross clamp (side clamp). The dark side of isolated root infusion of cardioplegia is represented by the potential inadequate overall distribution of cardioplegia during cardiac ischemic arrest [18,19,20]. Regarding strategies of proximal anastomosis, while the single cross-clamp strategy could prolong the total ischemic time, the side-clamp strategy on the other hand could cause a further residual ischemic time until the proximal anastomosis of vein grafts are completed. In patients with left ventricle depression, especially when a severe left main and/or multiple coronary stenosis are acknowledged, such a situation could potentially increase postoperative risk of poor outcome, and, therefore, alternative strategies to improve early recovery post cardiopulmonary bypass could be considered [21,22,23]. Here we analyzed the results of a combined strategy of total antegrade cold cardioplegia and total anterograde warm reperfusion (TAWR) to test the hypothesis that such an approach could be advantageous when compared with standard anterograde root cardioplegia and standard warm reperfusion (SAWR) in improving postoperative recovery in patients with left ventricle depression undergoing multi-vessel CABG.

## 2. Materials and Methods

In this observational, double-center, retrospective study, we compared the outcomes of patients with depressed left ventricle function undergoing coronary artery bypass graft (CABG) with two different strategies of myocardial protection. The inclusion criteria encompassed patients with depressed left ventricle function (ejection fraction < 40%) and multi-vessel coronary diseases necessitating CABG, including ≥2 vein grafts, elective timing for CABG and preoperative evidence of sinus rhythm without known arrhythmic abnormalities. The REDO CABG procedure and the need for combined mitral surgery were considered exclusion criteria.

Out of 97 patients who underwent, over a 5-year period, CABG using antegrade root cardioplegia combined with selective antegrade graft cardioplegia and total arterial warm reperfusion (TAWR), 32 met the inclusion criteria and were therefore enrolled as group A of the study. Early postoperative outcomes of patients in group A were compared to those of 32 matched patients meeting general inclusion criteria, undergoing CABG using the standard strategy of antegrade root cardioplegia and limited warm reperfusion during aortic side clamp for proximal vein graft anastomosis (SAWR) and enrolled as group B. Patients of the two group were matched based on personal data and diagnostic preoperative characteristics listed in Appendix A.

### 2.1. Detailed Cardioplegia Delivery Protocol

Following cardiopulmonary bypass (CPB) setup using standard aortic and right atrium cannulation plus root cannula for antegrade cardioplegia, CPB was started in mild hypothermia (34°). Once full CPB flow was achieved, the aorta was clamped and antegrade cardioplegia was infused from the aortic root.

Our standard infusion protocol is typically structured in two distinct phases (see Appendix B for the full protocol):

- ***Induction****:* Diastolic cardiac arrest is induced by a controlled blood flow of 300 mL/min on average, combined with cardioplegia infusion at a rate of 180–293 mL/h, with a target K+ concentration of up to 20–30 mEq/l. This process is maintained for at least 3 min.
- ***Maintenance (every 15–20 min or if there is evidence of a heartbeat):*** After the induction phase, myocardial protection is ensured by intermittent infusion of cardioplegia with maintenance of diastolic arrest by a blood flow of 200 mL/min, while the infusion rate of cardioplegia decreases to 45–83 mL/h. Potassium concentration is adjusted during this phase, which continues for at least 2 min, to 10–15 mEq/l.

This part of the protocol was identical in both study groups. The protocol then diverged according to the study group as follows.

### 2.2. Group A (TAWR)

**Figure 1:**
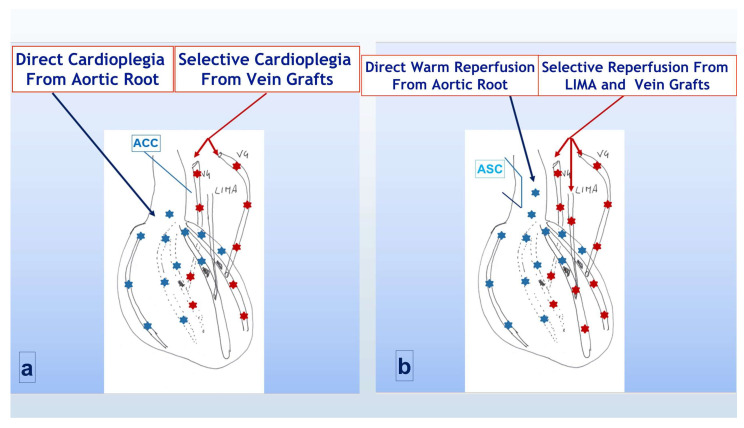
Cardioplegia and reperfusion strategy in group A (TAWR). (**a**) During aortic cross-clamp period; (**b**) following cross-clamp removal and side-clamp positioning. ACC = aortic cross clamp; LIMA = left internal mammary artery; VG = saphenous vein graft; ASC = aortic side clamp. Light blue asterisks indicated the flow through the native aortic root and coronaries, red asterisks indicated the flow through the CABG counduits.

In adjunct to the standard protocol, during maintenance, selective antegrade cold cardioplegia (using the some mEq/l of Kcl concentration) was also infused through vein grafts after each distal anastomosis was completed, thus extending the cardio-protection to the more peripheral district of the left ventricle, distal to the significant coronary stenosis (Figure 1a). Once distal anastomoses were completed (with the LIMA graft to LAD always being the last in both groups) before aortic cross-clamp removal, warm blood ri-perfusion of myocardial tissue distal to coronary anastomosis, through all vein grafts, was added to the standard distal reperfusion of LAD through the LIMA. Once the aortic cross clamp was removed and the aortic side clamp was positioned to complete proximal anastomosis, the warm reperfusion was then completed through the aortic root (Figure 1b), achieving what we called total antegrade cardioplegia and warm reperfusion (TAWR).

### 2.3. Group B (SAWR)

**Figure 2:**
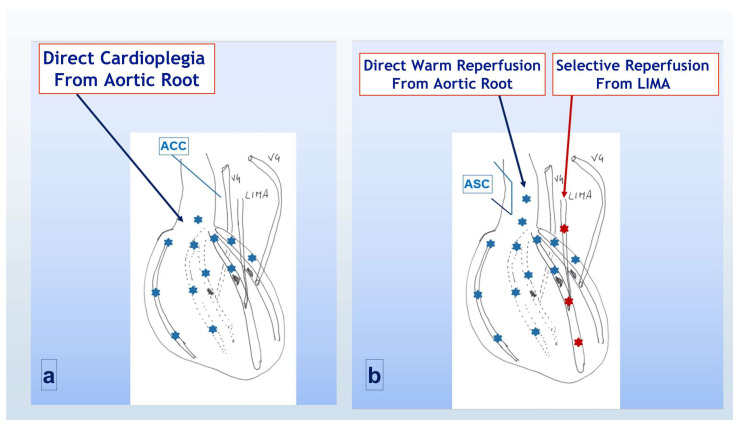
Cardioplegia and reperfusion strategy in group B (SAWR). (**a**) During aortic cross-clamp period; (**b**) following cross-clamp removal and side-clamp positioning. ACC = aortic cross clamp; LIMA = left internal mammary artery; VG = saphenous vein graft; ASC = aortic side clamp. Light blue asterisks indicated the flow through the native aortic root and coronaries, red asterisks indicated the flow through the CABG counduits.

Induction and maintenance were according to the standard protocol. During each maintenance dose cardioplegia reached, therefore, only the area of myocardium proximal to the coronary stenosis (Figure 2a). Once all distal anastomoses were completed, warm reperfusion was obtained, before aortic cross clamp removal, only distally to the LAD obstruction through the LIMA. Once the cross clamp was removed and the side clamp was positioned, warm reperfusion was also achieved proximally to the coronary stenosis through the antegrade flow (Figure 2b). With this strategy, which we called standard antegrade protection and warm reperfusion (SAWR), full perfusion of the distal myocardial area was delayed till all proximal anastomoses were completed, and the side cross clamp was finally removed.

To compare the efficacy of both strategies of myocardial protection, we considered as the primary endpoint the cumulative major outcomes (in-hospital death, peri-operative myocardial infarction or stroke); secondary endpoints were split as follows: (a) markers of intra-operative early cardiac recovery: spontaneous rhythm recovery; early hemodynamic recovery defined as the need for inotropic (dopamine/dobutamine infusion at >5 mcg/Kg/min or adrenaline infusion at >0.1 mcg/Kg/min) and/or mechanical (IABP, ECMO) support for CPB discontinuation; early postoperative left ventricle recovery assessed by trans-esophageal echo before sternal closure and (b) markers of postoperative late recovery: overall incidence of the prolonged need (>48 h) of inotropic and/or mechanical support (as previously defined), mechanical ventilation (>48 h) and cumulative ICU stay (>72 h), and late left ventricle recovery assessed by trans-esophageal echo at the patient’s discharge and comparison to pre-CPB LV global and regional function. Ultrasensitive troponin I level (TNI) was postoperatively monitored in all patients at 6, 12, 24, 48 h from CPB weaning and at discharge of the patient to rehabilitation. For the purpose of the study, the TNI value at 6 h, the peak value and the value at discharge were considered as they were recorded in the institutional database.

### 2.4. Statistical Analysis

All data were recorded in a designed database, and statistical analysis was performed using Medcalc software (Medcalc 18.2.1; Acacialaan 22, 8400 Ostend-Belgium). Normal distribution for the continuous variables was tested using the Kolmogorov–Smirnov test to optimize further the statistical test choice. Comparative statistics were performed, using a parametric (unequal variance, two-tailed *t*-test) or non-parametric test (Mann–Whitney for independent samples, Kruskal–Wallis) according to the results of Kolmogorov–Smirnov test to compare the continuous variables of the two study groups (*p* < 0.05 considered statistically significant). Comparison of the categorical variables was obtained with a chi-square analysis (Fisher exact test when appropriate) or a Mann–Whitney test (with *p* < 0.05 considered statistically significant). Data were expressed as mean ± sd or median/interquartile range according to normal distribution. Finally, multivariate analysis was used to detect independent risk factors for favorable early and late outcomes.

## 3. Results

As a result of patients matching, the two groups were homogeneous with respect to all preoperative characteristics, as summarized in Table 1.

Different myocardial protection strategies did not impact the characteristics of myocardial revascularization and surgical times, as ot impact ion anso s could expose to e single cross clamp time ardial protection during cardiac surgery procedursummarized in Table 2.

**Figure 3:**
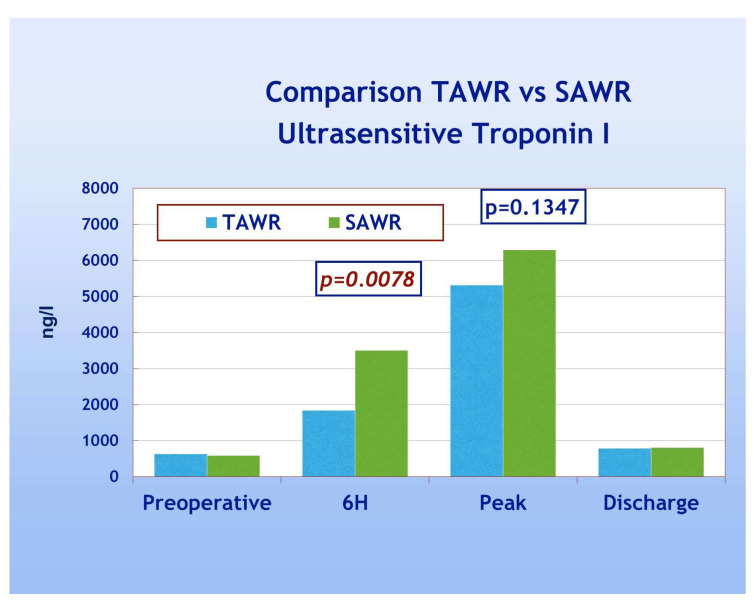
Postoperative TN1 trend TAWR vs. SAWR. TAWR: total antegrade cardioplegia and warm reperfusion; SAWR = standard antegrade cardioplegia and warm reperfusion; 6H: six hours following intensive care admission.

In looking at the primary endpoints, two patients died in-hospital (one from each group) with an overall in-hospital mortality of 3.15% and no significant differences between the two groups. In total, one patient (from group B) experienced a peri-operative myocardial infarction (according to troponin peak and echocardiography findings), and one patient (from group A) suffered from neurological complications with clinical and CT signs (stroke). Cumulative primary endpoints did not show, therefore, significant differences between the two groups. The overall trend of the release of TNI, on the other hand, showed significantly higher TNI release in group B (*p* = 0.0078) but only at 6 h from ICU admission (Figure 3). The peak release and value at discharge, conversely, did not show significant differences between groups. In the majority of patients the peak release was reached 12 h after CPB weaning in both groups, but a complete comparison between two groups in terms of exact time of peak release was not possible.

**Figure 4:**
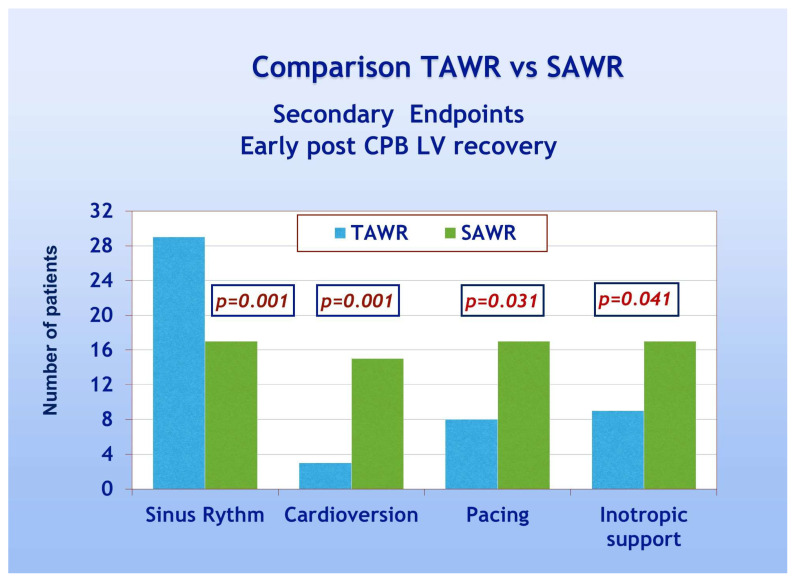
Early post-CPB LV recovery parameters: TAWR: total antegrade cardioplegia and warm reperfusion; SAWR = standard antegrade cardioplegia and warm reperfusion.

**Figure 5:**
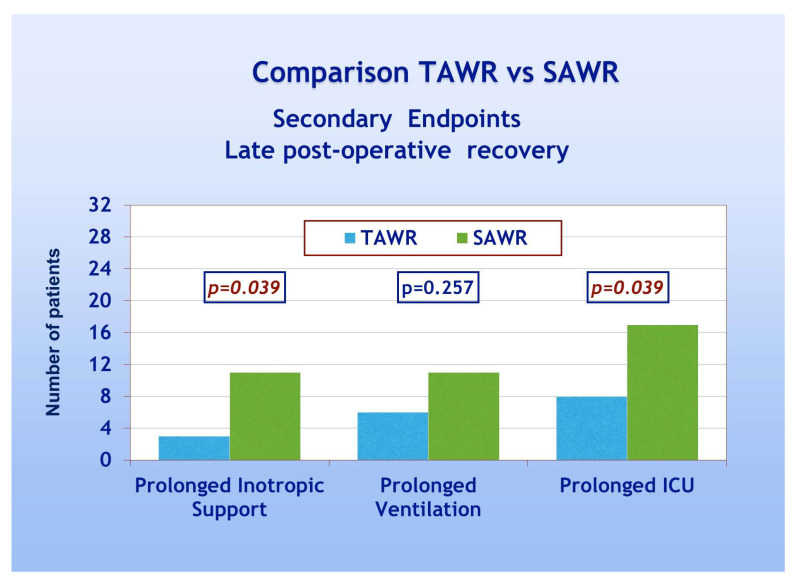
Late postoperative recovery: TAWR: total antegrade cardioplegia and warm reperfusion; SAWR = standard antegrade cardioplegia and warm reperfusion; ICU: intensive care unit.

With regard to secondary endpoints, Figure 4 shows a TAWR and SAWR comparison of parameters of early LV function recovery following CPB. Patients of group A showed significantly higher spontaneous recovery of sinus rhythm (90% and 53% for group A and B, respectively, *p* = 0.001). Patients of group A also showed significantly less frequent need for electrical cardioversion and pacing, thus confirming an overall faster recovery of spontaneous and regular electrical cardiac activity. Patients of group A, furthermore, showed significantly less frequent need for inotropic support compared to group B (28% and 53% for group A and B, respectively, *p* = 0.041). As far as the comparison between the two groups in terms of late postoperative recovery, as shown in Figure 5, according to previously defined criteria, patients of group A required significantly less frequently prolonged inotropic support and prolonged total ICU stay when compared to group B (*p* = 0.039). On the other hand, no significant difference was shown in the frequency of prolonged mechanical ventilation. As shown in Table 3, significant recovery of LVEF, compared to the preoperative condition, was shown in both groups either as early recovery (intraoperative trans-esophageal echo with inotropic support) or at discharge (pre-discharge trans-thoracic echo without inotropic support). No significant differences were shown between early and late recovery. The comparison between the two groups, however, never did reach statistical significance. Finally, multivariate analysis did not reach enough statistical power due to the low number of events.

## 4. Discussion

In this study we focused on the analysis of early clinical outcomes in patients with preoperative depressed left ventricle function undergoing coronary artery bypass graft surgery and receiving two different strategies of myocardial protection. Two peculiar aspects have to be considered in our study: (a) the selection of patients with preoperative left ventricle depression and (b) the choice of focusing the analysis on different strategies of myocardial protection rather than on the different composition of the cardioplegia used as the primary myocardial protection. Since the first randomized studies were published in 1980 [8], several further studies have focused instead on the comparison of different types of cardioplegia proposed to optimize postoperative outcomes (i.e., crystalloid cardioplegia vs. blood cardioplegia). From 2008 to date, several extensive meta-analyses, including a total of 70 randomized controlled trials, addressed such issues [10,14,15,16,17] without reaching a definitive answer. Postoperative mortality, the incidence of post-surgery myocardial infarction and low cardiac output were usually standard cut-offs for the majority of these studies, which rarely focused, however, on the strategy of cardioplegia infusion rather than on the type of cardioplegia. Isolate root infusion of cardioplegia could cause inadequate overall distribution, especially in the case of multiple severe coronary stenoses [18,19]. From a clinical point of view, optimizing the myocardial protection strategy should be extremely relevant, especially in patients undergoing high-risk surgical procedures, such as patients with depressed left ventricle function [20,21,22]. Retrograde cardioplegia infusion through a further catheter into the coronary sinus [23] has also been suggested in such conditions but, once more, some concerns have been raised due to the potential damage of the coronary sinus and also due to the potential inadequate protection of right heart [24]. Selective graft perfusion during coronary surgery, which was at the base of our study, is not itself an original idea as was reported back in the mid-1980s by Goldman et al. [25]. Selective graft perfusion is usually combined with a two-step aortic clamping strategy which includes distal anastomosis during a cross-clamp period and proximal anastomosis following cross-clamp removal and side-clamp positioning [26,27,28,29,30]. Such a strategy of myocardial revascularization should allow for a shorter overall ischemic time and a more comfortable measurement of vein graft length. Following its introduction, contrasting results in terms of favorable myocardial protection have been reported using selective vein graft cardioplegia infusion, and, therefore, this technique has not achieved worldwide diffusion [26,27,28]. Both negative [26,27] and positive [28] impacts of selective cardioplegia were shown in early studies, however, not including patients with LVEF < 30%. We designed this study moving from the concept that the routine protocol of antegrade root cardioplegia could limited cardioplegia diffusion in the area of the myocardium distal to severe coronary stenosis. In the two-step aortic clamping technique, furthermore, adequate antegrade flow distal to significant coronary stenosis is restored until the proximal anastomoses are also completed and vein grafts perfused only through the LIMA graft. Despite the evidence that in not-occluded coronaries a residual flow through the coronary is still maintained and allows for the first cardioplegic arrest, limited peripheral diffusion of isolated root cardioplegia, if compared to selective vein graft cardioplegia, should be considered [18,19]. During the side-clamp period, moreover, it is also proved that selective warm blood perfusion of vein grafts (which we called TAWR = total anterior warm reperfusion) allows for a more complete myocardial reperfusion if compared to the standard isolated distal reperfusion of the LAD territory (through LIMA). The purpose of our study was to evaluate whether this combined and comprehensive strategy (selective vein graft cardioplegic infusion + TAWR) not only allows for a more complete distal perfusion but could also promote enhanced early recovery of the left ventricle function following multi-vessel CABG surgery. To restrict the bias of patient selection, thus overcoming the primary limitation of previous studies using a similar approach [26,27,28], we limited the analysis to patients with known depressed left ventricle function (including patients with LVEF < 30%) and at least three coronary grafts. Looking at our primary endpoint, we could show no significant differences between the two groups in terms of mortality and major events, but we should stress that the number of overall events was quite low. Despite the overall incidence of postoperative acute myocardial event not being different between the two groups, the TNI release showed some differences. Conversely to data previously published [28], however, in our experience a combined approach allowed for a reduced TNI release but limited to 6 h postoperative. We could speculate that early postoperative TNI release should be a more reliable marker of better intra-operative myocardial protection compared to a late postoperative release. Our findings could confirm that the early reperfusion phase is of paramount relevance in myocardial protection following cardiopulmonary bypass and aortic cross clamp [16]. A single and repeated shot of blood/crystalloid cardioplegia seems to warrant better protection in terms of early and mid-term TNI release compared to single-shot long-protection cardioplegia [11,16]. Our experience seems to support such an idea as reduced TNI release was not reported using different cardioplegias and techniques of infusion [14,20]. The analysis of secondary endpoints, on the other hand, showed further interesting findings. As previously described, we split our analysis between early and late recovery, and in both cases we found significant differences. Better early recovery using TAWR was confirmed by the higher incidence of spontaneous sinus rhythm recovery and the lower incidence of the need for electrical defibrillation, pacing and inotropic support following cross-clamp removal. Such a strategy, therefore, could overcome the increased incidence of arrhythmia reported following CABG using cold cardioplegia (regardless of the type of cardioplegia) [31]. Although spontaneous recovery of the sinus rhythm could be considered a debatable parameter of better myocardial protection, it has been widely considered in such a respect in previous studies [28,29,30]. As far as late recovery, despite inotropes use following cardiac surgery always being a troublesome parameter to evaluate, we showed a clear trend in the reduced need for prolonged inotropes and reduced ventilation time and overall ICU stay in patients operated on using TAWR strategy. Our study, therefore, seems to support recent reports focused on specific patient subgroups, revealing promising results in terms of early LV recovery following CABG surgery using such a strategy [29,30]. Surely a more conspicuous study population would have been beneficial in elucidating such aspects as the relevance of the number of patients in limiting the significance of many studies addressing the advantages of a peculiar type of myocardial protection has been previously underlined [32,33]. Early and late recovery of echocardiographic LV function, using the TAWR strategy, deserves a focused comment. The increased LVEF at the end of CPB in the TAWR group of our experience was not consistent with previous data reported for CABG in patients with depressed preoperative LVEF [20,22] regardless of the type of cardioplegia used. The early postoperative phase, however, is surely a very delicate surgical moment and several factors could also contribute to the different measurements of LVEF (i.e., dose and timing of inotrope infusion). Late recovery in terms of LVEF at discharge, however, reflects clear satisfactory results of the surgical procedure, including myocardial protection during the ischemic period. In our experience LVEF increases in both groups of patients, and despite a positive trend in the TAWR group, there is no statistical difference between the two groups. Once more, limitation of the patient population could have played a relevant role in this respect. In conclusion despite several studies over the last decades [14,15,17] having focused on elucidating the best option for myocardial protection during cardiac surgery procedures, clear evidence of the superiority of a single type of cardioplegia is still lacking, and the surgeon’s own preference is still the key factor in surgical choice [34]. Non-uniform management of either the study design or surgical protocol is a common bias of the majority of these studies, along with the suggested evidence that the technique of infusion of cardioplegia and reperfusion is as relevant as the type of cardioplegia itself. Our final comment is related to what has to be considered the key point of our study: the simple comparison of myocardial protection strategy focused on the different modality of cardioplegia infusion and reperfusion in a standard setup. In this respect our study seems to give, in our mind, interesting findings on the potential advantages of a well-known old-style method of myocardial protection in patients with severe multi-vessel diseases and depressed LV function undergoing CABG and could be used as a starting point for further studies based on a more extensive application of this strategy.

## 5. Conclusions

In conclusion our study seems to suggest that selective graft cardioplegia and anterograde total reperfusion could significantly contribute to enhancing early postoperative cardiac recovery in patients with poorly functioning left ventricles undergoing multi-vessel CABG. The hypothesis that the TAWR technique could be beneficial in reducing post-cross-clamp removal ischemia compared to isolated standard root anterograde cardioplegia deserves in our mind further and more extensive studies, possibly in the context of a multicenter randomized trial.

## 6. Limitations

This study has some relevant limitations. First of all, as clearly mentioned in the text, it has not considered a comparison between different types of cardioplegia but only between different strategies of cardioplegic infusion and coronary reperfusion. We also already acknowledged in the text that this study does not consider the strategy of retrograde cardioplegia infusion and reperfusion through the coronary sinus. It should also be mentioned and acknowledged that the TAWR strategy does not enhance myocardial protection into the area served by the LAD unless a vein graft is also used on the LAD. Finally, the limited number of patients and, therefore, of major events reduces the statistical power of the comparison.

## Figures and Tables

**Table 1:** Preoperative data: TAWR: total antegrade warm reperfusion; SAWR: standard antegrade warm reperfusion. MI: myocardial infarction; PCI: percutaneous coronary interventions; LVEF: left ventricle ejection fraction; PAPs: pulmonary artery pressure (systolic).

**Personal Data**	**Group A** **TAWR (n.32)**	**Group B** **SAWR (n.32)**	***p***
Gender			0.5452
Male	24 (75)	26 (81)	
Female	8 (25)	6 (19)	
Age			0.704
Mean ± sd (years)	68 ± 10	67 ± 11	
>75 years	15 (47)	15 (47)	
**Comorbidities**	**Group A**	**Group B**	***p***
Diabetes	18 (56)	17 (53)	0.803
Hypertension	21(65)	22 (68)	0.788
Smoking History	20 (62)	19 (59)	0.797
Hypercholesteloremia	19 (59)	20 (62)	0.797
COPD	11(34)	10 (31)	0.795
Chronic Renal Failure	9 (28)	10 (31)	0.784
**Cardiac Parameters**	**Group A**	**Group B**	***p***
Previous MI	20 (62)	18 (56)	0.601
Previous Stent-PCI	16 (50)	11 (34)	0.205
LVEF			
Mean ± sd	33 ± 4	34 ± 5	0.542
<30%	9 (28)	8 (25)	0.777
Mitral Regurgitation			
Trivial	8 (25)	9 (28)	0.777
Moderate	3 (9)	2 (6)	0.642
Aortic Regurgitation			0.688
Trivial	4 (12)	3 (9)	
Moderate	0	0	
PAPs > 40mmHg	3 (9)	2 (6)	0.642

**Table 2:** Operative data. IMA: internal mammary artery; ECC: extracorporeal circulation; TAWR: total antegrade warm reperfusion; SAWR: standard antegrade warm reperfusion.

Operative Parameters	Group A TAWR (n.32)	Group B SAWR (n.32)	*p*
Number of Grafts	3.5 ± 0.6	3.4 ± 0.5	0.37
Single IMA Double IMA	30 (94) 2 (6)	31 (97) 1 (3)	0.98 0.97
Surgical Time (min)	270 ± 66	256 ± 80	0.48
ECC Time (min)	104 ± 15	103 ± 11	0.65
Cross-Clamp Time (min)	70 ± 10	68 ± 10	0.69
Side-Clamp Time (min)	27 ± 7	26 ± 4	0.56

**Table 3:** Postoperative left ventricle function recovery in group A vs. group B. LVEF: left ventricle ejection fraction; TTE: trans-thoracic echocardiography; TEE: trans-esophageal echocardiography; TAWR: total antegrade cardioplegia and warm reperfusion; SAWR = standard antegrade cardioplegia and warm reperfusion. °: with inotropic support. * means that this value get a significant difference with the corresponding value at T0.

Group	T0 Preoperative (TTE LVEF)	T1 Intraoperative ° (TEE LVEF)	T2 Discharge (TTE LVEF)	*p*
TAWR	33 ± 4%	38 ± 3% *	40 ± 3% *	0.001 * Vs. T0
SAWR	34 ± 5%	37 ± 5% *	39 ± 4% *	0.001 * Vs. T0
*p*	0.542	0.55	0.18	

## Data Availability

Data cannot be shared as such for policy reason. As it has been specified Data are stored and upon specific request could be shared.

## Abbreviations

CABG: coronary artery bypass graft, TAWR: total antegrade warm reperfusion, SAWR: standard antegrade warm reperfusion, CPB: cardiopulmonary bypass, LIMA: left internal mammary artery: LVEF: left ventricle ejection fraction, IMA: internal mammary artery, ECC: extracorporeal circulation.

## Appendix A

Parameters included in patients matching:

**Personal Data**	**Preoperative Evaluation**
Age	LVEF
Gender	Degree of MR
	AV Regurgitation
	PAP
**Medical History**	**Risk Factors**
Previous MI	Hypertension
Previous PCI	Hyperlipidemia
Reason for CABG	Diabetes
Combined Mitral Regurgitation	
Multivessel Coronary Diseases	
COPD	
CRF	
MI: myocardial infarction; PCI: percutaneous cardiac intervention; COPD: chronic obstructive pulmonary disease; CRF: chronic renal failure; LVEF: left ventricle ejection fraction; MR: mitral regurgitation; AV: aortic valve; PAP: pulmonary artery pressure.	

## Appendix B

Flow protocol for blood cardioplegia.

								**[K+]**	**CPL**
**Blood Flow**		**[K+] = mEq/l**	**[K+] = mEq/l**	**[K+] = mEq/l**	**[K+] = mEq/l**	**[K+] = mEq/l**			
		10	15	20	30	40			
50	mL/min	11	21	30	4	68	mL/h		
100	mL/min	23	41	60	98	135	mL/h		
150	mL/min	34	62	90	146	203	mL/h		
200	mL/min	45	83	120	195	270	mL/h		
250	mL/min	56	103	150	244	338	mL/h	Solution for Cardioplegia Flow	
300	mL/min	68	124	180	293	405	mL/h	K+ = 2 mEq/mL + Mg = 2 mEq/mL	
350	mL/min	79	144	210	341	473	mL/h		
400	mL/min	90	165	240	390	540	mL/h		
450	mL/min	101	186	270	439	608	mL/h		
500	mL/min	113	206	300	488	675	mL/h		
Details of the cardioplegia infusion protocol during (a) induction (red font and light blue background) and (b) maintenance (green font and light grey background). During induction, the standard target K+ concentration is 20–30 mEq/l, achieved with 300 mL/min of blood flow mixed with 180-293 mL/h of a solution of 2mEq/ml Potassium Chloride and 2mEq/ml of Magnesium Chloride Exaidrate. During maintenance, the standard target K+ concentration is 10–15 mEq/l, achieved with 200 mL/min of blood flow mixed with 45–83 mL/h of the same solution of Potassium and Magnesium.									
